# A review on functional ingredients in red meat products

**DOI:** 10.6026/97320630015358

**Published:** 2019-05-15

**Authors:** Tahreem Kausar, Entesar Hanan, Omeera Ayob, Bushra Praween, Zraa Azad

**Affiliations:** 1 Department of Food Technology, School of Interdisciplinary Sciences and Technology, Jamia Hamdard, Hamdard Nagar, New Delhi-62, India

**Keywords:** Enrichment, functional ingredients, meat, processing, value addition

## Abstract

Meat and meat products are important foods with essential nutritional components such as essential amino acids, fatty acids, vitamins and minerals that form a significant component for the normal physiological and biochemical processes. However, the main drawback of meat and meat products is the absence of dietary fibre and the presence of saturated fat. Value improvement can be done by the incorporation of functional ingredients into the meat products. The use of functional ingredients in meat products offers processors the opportunity to enhance the functional and nutritional value of their products. Vegetable proteins, dietary fibre, cereal by-products, fruits, legumes, spices, herbs, and lactic acid bacteria that have been used alone or in combination for the enhancement of the functional value of meat and meat products were studied. Hence, the current review focuses on the functional ingredients incorporated in meat products

## Background

Foods are important and suitable vehicles for the human being to convey the essential nutrients that may improve their health. Animal meat is of high biological value and a good source of proteins in many countries. Meat and meat products are ideal sources of soluble minerals, vitamins, essential fats, amino acids and many other nutrients having a specific function to the body 1. There is an increasing demand for healthier meat and meat products containing low levels of fat, cholesterol, reduced content of sodium chloride and nitrite, updated fatty acid profile composition and added health-enhancing ingredients among consumers worldwide. Recently, there is an increasing concern about health-oriented functional meat products as a result of drawbacks incorporated with muscle foods and its related health hazards. Meat is a good source of omega-3 fatty acids, proteins, vitamin B12 and high levels of iron 2. Meat and meat products can be modified by the addition of certain ingredients that eliminate or reduce harmful components from the body and are thus beneficial to health. Meat products incorporated with dietary fibres are best meat substitutes because of their functional and nutritional values 3. Goat meat is the most staple red meat taken in human diets. It is universally accepted but influenced by traditions and socioeconomic conditions; as a result, influencing customer preference 4, 5. Goat meat and beef were also slightly more liked due to preferences for texture and muscles. Goat meat is dark red with a coarse texture and has a noticeable variety of flavour and aroma from lamb and beef 6, 7. According to the consumer, meat and meat product consumption is unhealthy due to the presence of cholesterol, synthetic antioxidants; antimicrobial contents that result in some degenerative diseases and saturated fats 8.

**Figure 1:**
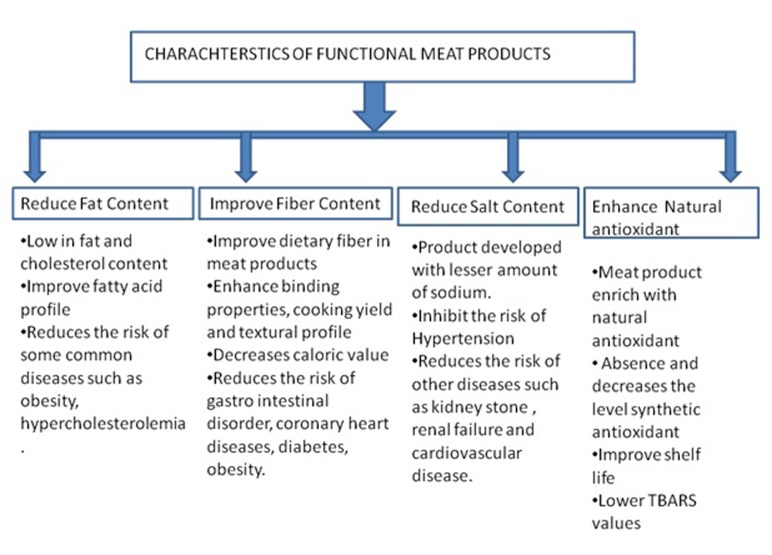
Characteristics of functional meat products

Functional food is mainly a conventional food, which is consumed as a part of a usual diet. The term functional food was firstly used by Japan in the 1980s to dedicate food products that are fortified with specific constituents with beneficial physiological effects 9. Foods that are marketed under functional category contain added technologically developed ingredients 10, and important biologically active compounds 11. These foods provide health benefits by mediating specific physiological functions in the body and are marketed and consumed for this value-added property. Reformulation of meat is achieved by the addition of fibres, proteins, polyunsaturated fatty acids (PUFA), antioxidants, etc. A functional food should possess certain requirements, i.e. it should be derived from a naturally occurring ingredient; consumed as a part of a regular diet and should be involved in the regulation of certain human process such as age delaying, risk prevention from diseases and improvement in immunological abilities 12. Sometimes in functional foods, one or more additional ingredients are added, that shows health benefits above and beyond as compared to those of regular foods 13. Functional food is widely used in developed countries due to their high shelf life, advanced food technology, health benefits, and known importance. Food nowadays is not only eaten for hunger satisfaction and necessary nutrition but also for preventing nutrition-related disorders and for mental-physical well being of an individual 14. Hence, food nutritionist and technologist are targeting to develop functional meat products with great efforts that possess natural antioxidants and antimicrobials, low fat, lesser sodium content, enriched with dietary fibres and ω-3 and ω-6 fatty acids 15 as shown in Figure 1.

## Incorporation of dietary fiber:

**Figure 2:**
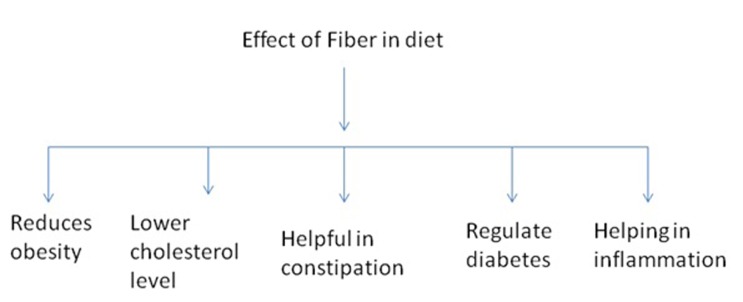
Effect of Fiber in diet

The plant-based derivatives like fruits, nuts, vegetables, herbs, and spices are mainly used now a day for the production of modified and healthier meat products with improved shelf life. Dietary fibers and antioxidants addition are the most approaching step in the development of novel meat products. The fiber incorporation is on demand because of its technological use and benefits to human health 16. Foods with high dietary fiber proportion are reported to reduce the risk of obesity, colon cancer, cardiovascular diseases and various other disorders 17 as shown in Figure 2. Various dietary fibers have been used in meat products for determination of proper beneficial health effects and also as potential fat substitutes 18.

Dietary fibers (DF) are defined as residues of eatable plant fragments and carbohydrates that can't be absorbed or assimilated and are indigestible in the small intestine of human 19. It promotes important physiological effects such as laxation and blood glucose and cholesterol attenuation 20. Dietary fibers are composed of various categories of ingredients namely oligosaccharides and polysaccharides, i.e., cellulose, hemicelluloses, pectic materials, inulin, lignin and other components like waxes, phytates, cutin, saponins, and resistant proteins and polyphenols 21. Various DF sources are wheat, oat, and rice bran; sugar beet; soy; brewer's spent grain; pea; vegetables; cereal grains; woody plants; fruits; legumes; leguminous plants, psyllium, etc. have been incorporated in the recipes of certain meat products namely meatballs, patties, and sausages for nutritious daily regime improvement. DF incorporation in meat products improves functional properties such as water retention, lubrication, rheological properties, emulsion stability, neutral flavor and modification in texture 22. It also improves cooking yield, reduces formulation costs and enhances the palatability. Dietary Fiber intake through meat substituted with fruits, vegetables, and certain grains protects against cardiovascular diseases, diverticulitis, constipation, irritable colon, colon cancer and diabetes 23. Dietary fibers also act as a fat replacer. It also decreases plasma and LDLcholesterol levels, reduces the risk of dietary related problems like obesity, coronary diseases, gastrointestinal disorders, i.e., constipation, inflammatory bowel diseases, etc 24. Various food industries manufacture modified energy-dense foods by adding vegetable and fruit fibers. Fiber addition in meat products is considered as suitable and thus increases the cooking yield and texture in cooked meat products because of its water and fat binding properties 12. Supplementation of DF increases the bulk and reduces cooking losses in meat products. It offers no or fever changes in textural parameters by improving water-binding abilities and also enhances economic advantages for the consumers and processors 25. The dietary fiber in meat products is mainly considered clinically better as compared to that of traditional meat products 22. Also, the incorporation of dietary fiber in meat products leads to the development of novel meat products. Various types of dietary fibers improve the quality of the meat products are given in Table 1.

## Addition of antioxidants:

Meats and meat products are mainly prone to deterioration as they are rich in nutritional composition 37 and moisture content 38. Addition of spices, herbs, and vegetables extract to raw and cooked meat enhance total antioxidant capacity that is considered important criteria for the shelf life of the meat products, decrease lipid oxidation and improve colour stability 14. Lipid oxidation decreases food's nutritional quality due to loss of essential fatty acids and vitamins and results in a toxic reaction in the muscles foods such as malonaldehyde (MDA) and COPs, i.e. cholesterol oxidation products 39, 40. There are various methods for controlling lipid oxidation, among which use of natural antioxidants are the most reliable, effective, convenient and economical. An antioxidant such as herb stabilizes food lipids and thereby inhibits the quality deterioration of the products and also increases the shelf life of the products. Herbs also reduce risk of diseases and promote healthy well being due to their role to protect the body against oxidative damage. Antioxidants are those substances which, when present in food or the body at low concentrations delays or prevent an oxidative process that leads to quality deterioration in food and initiates degenerative diseases in the body. Lipid oxidation may occur via auto-oxidation, photooxidation, thermal oxidation, and enzymatic oxidation and mostly involve free radicals and other reactive species as the intermediate 41. Auto-oxidation is the reaction between atmospheric oxygen and lipids and is one of the common causes behind the oxidative deterioration of food and biological systems. The thermal oxidation refers to the process that can be accelerated at higher temperatures as experienced during deep fat frying. It leads to elevated levels of free fatty acids and polar matters, foaming, color, and viscosity. The process that involves photo sensitizer excitation and transfer of energy to lipid molecules or oxygen are mainly referred to as photo-oxidation. Enzymes, such as lipoxygenases also catalyze fatty acid oxidation and are usually inactivated in thermal processing of food. Natural antioxidants are derived from plants, animals and microorganisms and synthetic materials from chemicals. Plants and their constituents are natural inhabitants of antioxidants like tocopherols and polyphenols found mainly in spices, herbs, fruits, vegetables, cereals, grains, seeds, teas, and oils. Some antioxidants are also of marine origin, i.e., from algae, shellfish and marine bacteria 42, 41. By-products obtained from the food and agricultural industries have been reported for their potential use as antioxidants like hulls; shells and skins of nuts and cereals; citrus peels and seeds; canola meal. Fish viscera extracts have also been found to possess antioxidant activity 43. Synthetic antioxidants are derived from chemicals namely butylated hydroxyanisole (BHA), butylated hydroxytoluene (BHT), propyl gallate (PG) and tertiary-butyl hydroquinone (TBHQ) has been extensively used as food preservatives because of their low cost and bland flavor. Primary oxidation of lipid in meat products leads to cardboard flavor and with progress results in the development of rancid and oxidized flavor 44. Antioxidant-rich sources are mainly fruits and vegetables 45, 46 and serve as a natural source of antioxidants in meat products. It contains water-soluble vitamin such as ascorbic acid, flavonoids as well as fat-soluble vitamins and precursors like tocopherols and carotenoids. Antioxidants are chemical compounds that donate hydrogen to the free radicals and reduce rancidity and delayed lipid per-oxidation without altering sensory or nutritional properties of meat products 47. It has been reported that in last few years for meat product preservation butylated hydroxyanisole (BHA), butylated hydroxytoluene (BHT) and tertiary butyl hydroquinone (TBHQ) have been used as a synthetic antioxidant 48. However, synthetic chemicals used as an antioxidant often result in adverse effects on human health. So, the consumers are interested in the natural source of antioxidant for the application in meat products. Many studies reveal the addition of natural antioxidant in meat products improved the antioxidant properties and colour stability, reduced lipid oxidation, which improves the shelf life of the products (Table 2).

## Conclusion

Meat and meat products are good sources of protein that suffices the requirement of an individual. In order to enhance the nutritional quality, texture, flavour, color and shelf life of the meat products the inclusion of functional ingredients is of paramount importance. The incorporation of the functional ingredient in meat products reduces the possibilities of risk to chronic diseases. The increasing concept of using food for health benefits along with nutrition gives new opportunity to the meat industry. The addition of dietary fibre and natural herbs, which serve as a potential source of fibre and anti-oxidants respectively, fulfills the demand of the consumers for foods with functional value. Hence, the known functional ingredients in meat products are summarized in this report.

## Conflict of Interest

Authors declare no conflict of interest.

## Figures and Tables

**Table 1:** Functional ingredients as fiber content in meat products

S. No	Functional ingredients	Developed Product	Effect on meat product quality	Reference
1	Pumpkin	Chicken Sausages	Fiber enrich product	[26]
2	Psyllium husk	Chicken burger patties	Improve Dietary fiber content and reduces fat cholesterol content	[27]
3	Flax seed oil and rice bran	Beef burger patties	Diminish total lipid and saturated fatty acid	[28]
			Improve dietary fiber	
4	Carrot and Lemon Fiber	beef hamburger	Low fat and cholesterol content	[29]
5	Green banana and soybean hulls flours	Chicken nuggets	Improve dietary fiber and boost instrumental texture and color properties	[30]
6	Guava	Sheep Meat Nuggets	Improve antioxidant and dietary fiber	[31]
7	Psyllium husk and fenugreek leaves	Goat meat patties	Improve fiber content and antioxidant properties	[32]
8	Glutinous rice flour	Beef patties	Improve texture quality	[33]
9	Flax seed and tomato paste	Beef patties	Improve fatty acid profile and nutritional properties	[34]
10	Carrots and Oats	Chicken meat cutlet	Higher moisture, lower free fatty acid	[35]
11	Finger Millet Flour (eleusinecoracana)	Chicken patties	Improve cooking yield, and moisture retentions.	[36]

**Table 2:** Functional ingredients as a natural antioxidant in different types of meat products

S.N	Functional ingredients	Developed Product	Effect on meat product quality	Reference
1	Aqueous extract curry leave and fenugreek leaves	Raw chicken meat products	Improve antioxidant activity	[49]
2	Clove powder	Chicken patties	Anti microbial properties	[50]
3	Pomegranate rind powder	Chicken patties	Antioxidant potential	[51]
4	Guava	Sheep�Meat Nuggets	Improve antioxidanrt and dietryfiber	[52]
5	Tea polyphenol	Pork sausages	Antioxidant antimicrobial properties,Inhibit TBARs value	[53]
7	Rosemary extract	Raw and precooked pork sausages	Delay TBARS value	[54]
8	Extracts of kinnow rind, pomegranate rind and seed powders	Goat Meat Patties	Antioxidant	[55]
9	Fenugreek Seed Flour	Beef burger	Antioxidant and Antimicrobial properties	[56]
11	Broccoli powder extract	Goat meat products	Antioxidant�effect	[57]
12	Ground mustard	Chicken nuggets	Lower TBARS value, anti microbial properties	[58]
